# Alterations in Faecal Metagenomics and Serum Metabolomics Indicate Management Strategies for Patients With Budd-Chiari Syndrome

**DOI:** 10.3389/fcimb.2021.730091

**Published:** 2021-10-21

**Authors:** Qinwei Lu, Hao Xu, Lin Zhou, Ruifang Zhang, Zhen Li, Peng Xu, Tao Bai, Zhiwei Wang, Gang Wu, Jianzhuang Ren, Dechao Jiao, Yan Song, Rongtao Zhu, Jian Li, Weijie Wang, Ruopeng Liang, Lin Li, Xiuxian Ma, Maoheng Zu, Yuling Sun

**Affiliations:** ^1^ Department of Hepatobiliary and Pancreatic Surgery, The First Affiliated Hospital of Zhengzhou University, Zhengzhou, China; ^2^ Institute of Hepatobiliary and Pancreatic Diseases, Zhengzhou University, Zhengzhou, China; ^3^ Department of Interventional Radiology, The Affiliated Hospital of Xuzhou Medical University, Xuzhou, China; ^4^ Department of Digestive, The First Affiliated Hospital of Zhengzhou University, Zhengzhou, China; ^5^ Department of Ultrasound Diagnosis, The First Affiliated Hospital of Zhengzhou University, Zhengzhou, China; ^6^ Department of Endovascular Surgery, The First Affiliated Hospital of Zhengzhou University, Zhengzhou, China; ^7^ Department of Interventional Radiology, The First Affiliated Hospital of Zhengzhou University, Zhengzhou, China; ^8^ Department of Vascular Surgery, The First Affiliated Hospital of Zhengzhou University, Zhengzhou, China

**Keywords:** Budd-Chiari syndrome, gut microbiota, metagenomics, metabolomics, management strategies

## Abstract

We investigated the effects of gut microbiota and serum metabolite levels in patients with Budd-Chiari syndrome (B-CS) and their importance for guiding clinical management strategies. In total, 214 B-CS patients (93 untreated and 121 treated) and 41 healthy controls were enrolled. Gut microbiota and serum metabolome were analysed using shotgun metagenomics and liquid chromatography-mass spectrometry. The gut microbiota of the patients showed abundance of *Campylobacter* and low levels of *Saccharomyces*, *Deinococcus*, and *Thiomonas* (P < 0.05). Thirty metabolites, including taurocholate and (R)-3-hydroxybutyric acid, were identified in the patients (VIP > 1, P < 0.05 and FC > 1.2 or FC < 0.83). Random forest (RF) models showed that serum metabolome could effectively identify B-CS from healthy controls and RF-metabolomics exhibited perfect discrimination (AUC = 100%, 95% CI: 100% – 100%), which was significantly higher than that achieved by RF-metagenomics (AUC = 58.48%, 95% CI: 38.46% – 78.5%). *Campylobacter concisus* and taurocholate showed significant positive correlation in patients with clinical manifestations (P < 0.05). *Actinobacteria* levels were significantly higher in untreated patients than in treated patients (P < 0.05). *Campylobacter* and *Veillonella* levels were significantly higher in treated patients than in healthy controls (P < 0.05). We identified major alterations in the gut microbiota and serum metabolome of patients with B-CS. Faecal metagenomics- and serum metabolomics-guided management strategies are required for patients with B-CS.

## Introduction

Budd-Chiari syndrome (B-CS) refers to partial or complete obstruction of the hepatic venous outflow tract in the absence of cardiac and pericardial disease (DeLeve et al., 2009). The incidence of B-CS is associated with multiple risk factors, including myeloproliferative disorders, pregnancy, antiphospholipid syndrome, use of coagulants, and antithrombin III protein C and S deficiency in Western countries (Patel et al., 2006; Parkash et al., 2017; Obed et al., 2018; Iliescu et al., 2019). However, studies on B-CS in Asian countries are limited.

Owing to its insidious onset, clinical features are almost normal in the early stages of B-CS. Portal hypertension (PHT) and inferior vena caval hypertension (IVCHT) are the primary manifestations in the middle and late stages of B-CS (Bhatt et al., 2019). Patients often present with hypertension, hypersplenism, and cirrhosis, and, in severe cases, sudden death (Valla, 2002). Previous studies have shown that evaluation *via* imaging [ultrasound and enhanced computed tomography (CT) scan or magnetic resonance imaging (MRI)] plays an important role in the diagnosis of B-CS, which shows occlusion or compression of hepatic veins and/or retrohepatic veins and venous collaterals, along with morphological changes in the liver with caudate lobe hypertrophy and delayed nodule formation (Bansal et al., 2018; Van Wettere et al., 2018). Asymptomatic B-CS is associated with spontaneously formed intrahepatic and portosystemic venous collaterals (Hadengue et al., 1994). Currently, the identification of B-CS and management strategies has attracted significant attention. Unfortunately, randomized clinical trials comparing the different treatment regimens for B-CS are still lacking. Based on clinical experience, retrospective studies, and expert consensus, the current treatment strategy for B-CS relies on progressively increasing invasiveness to correct hepatic venous outflow obstruction, although its efficacy has not been evaluated in detail (Hernandez-Gea et al., 2019). Therefore, the development of new non-invasive diagnostic methods, timely assessment of the condition, and guidance of current management strategies are crucial.

Alterations in gut ecosystems are associated with changes in human health and disease. Our previous study reported an association between intestinal bacteria and B-CS. Correction of gut microbial alterations may be considered a potential strategy for B-CS prevention and treatment (Sun et al., 2018). High-throughput sequencing technologies and a suite of computational pipelines have been incorporated into shotgun metagenomics approaches, which have changed microbiological research (Quince et al., 2017). In addition, metabolomics is an emerging approach that follows genomics and proteomics, involving high-throughput identification and quantification of the metabolome (Jang et al., 2018). Based on the continuous development of advanced bioinformatics, metabolomics as a systematic diagnostic tool has been widely used in biomedical and clinical research (Tang et al., 2011; Bonvallot et al., 2014). To our knowledge, metabolomics have not been applied to identify new biomolecular features of B-CS.

Here, we aimed to characterise the microbiota of Chinese patients with B-CS using metagenomic shotgun sequencing and compare them with those of healthy (control) individuals. To better understand the metabolism of B-CS, we detected changes in serum metabolites using UHPLC-QTOF-MS. Our study provides possible explanations for the differences observed among different phenotypes of B-CS and provides new perspectives regarding invasive treatment of B-CS.

## Methods

### Patients and Samples

We prospectively established two unique independent cohorts from the First Affiliated Hospital of Zhengzhou University and the Affiliated Hospital of Xuzhou Medical University, China. Patients (from inpatient and outpatient departments) in the cohort were diagnosed with Budd-Chiari syndrome (B-CS) using a multidetector computed tomography (CT) three-dimensional vascular reconstruction technique combined with colour Doppler ultrasound. Neither the patients nor healthy controls used antibiotics in the past 3 months without taking probiotic preparations or dietary yogurt for 1 month. In particular, none of the subjects suffered from any disease affecting intestinal microorganisms, including obesity, hypertension, diabetes, intestinal diseases, long-term diarrhoea, and constipation. All healthy controls were family members of patients with B-CS who shared the same diet and living environment. All participants provided written informed consent for participation in the study. The study protocol was approved by the local ethics committee.

Blood samples from all individuals were collected in 5 mL EP tube, then centrifuged at 4,000 rpm for 10 min at 4°C to extract serums to new Eppendorf tubes and stored at -80°C for further detection. A total of 100 μL of each thawed sample was placed in 2 mL EP tubes and extracted as previously described (Dunn et al., 2011). Then, 75 μL supernatant was transferred into a 2-mL liquid chromatography-mass spectrometry (LC/MS) glass, and 10 μL of each sample supernatant was pooled as quality control (QC) samples. Finally, a 75 μL supernatant was used for the UHPLC-QTOF-MS analysis.

Similarly, fresh faeces samples were collected in 5-mL EP tubes and stored at -80°C for further shotgun metagenomics sequence. Microbiota DNA was extracted at Novogene Bioinformatics Technology (Beijing, China) using the sodium dodecyl suplphate (SDS) method. DNA concentration and purity were assessed using 1% agarose gels, and DNA was subsequently diluted to 1 ng/μL using sterile water. DNA degradation degree and potential contamination were monitored on 1% agarose gels. The NanoPhotometer^®^ spectrophotometer (IMPLEN, CA, USA) was used for DNA purity (OD260/OD280 and OD260/OD230) detection. The Qubit^®^ dsDNA Assay Kit in Qubit^®^ 2.0 Fluorometer (Life Technologies, Carlsbad, CA, USA) was used for DNA concentration measurements.

### UHPLC-QTOF-MS Analysis

LC-MS/MS analysis was performed using an Agilent 1290 Infinity UHPLC system coupled to a Triple TOF 6550 (Agilent Technologies) to acquire the full scan MS1 data, a Triple TOF 6600 mass spectrometer (AB Sciex) to acquire MS/MS spectra, and an UPLC BEH Amide column (2.1 * 100 mm, 1.7 μm, Waters) (Zhang et al., 2019). The mobile phase was composed of 25 mmol/L ammonium acetate and 25 mmol/L ammonia hydroxide in water (pH = 9.75) (A) and acetonitrile (B). The analysis elution gradient was as follows: 0 ~ 0.5 min, 95% B; 0.5 ~ 7.0 min, 95% ~ 65% B; 7.0 ~ 8.0 min, 65% ~ 40% B; 8.0 ~ 9.0 min, 40% B; 9.0 ~ 9.1 min, 40% ~ 95% B; 9.1 ~ 12.0 min, 95% B. The column temperature and auto-sampler temperature were set to 25°C and 4°C respectively. The injection volumes were 2 μL for both positive and negative modes. The parameters for full scan MS1 data acquisition in 6550 QTOF mass spectrometry (Agilent Technologies) were set as follows: the scan range was 60 – 1200 Da; gas temperature, 250°C; gas flow, 16 L/min; sheath gas temperature, 350°C; sheath gas flow, 12 L/min; nebulizer, 20 psi; fragmentor, 175 V; capillary voltage, 3,000 V, were set as the electrospray ionisation (ESI) source conditions. MS/MS spectra was obtained in an information-dependent basis (IDA) mode in Triple TOF 6600 mass spectrometer (AB Sciex) with the acquisition software (Analyst TF 1.7, AB Sciex) with the following criteria: 12 precursor ions with intensity above 100 were selected and then fragmented in each cycle (0.56 s) under the collision energy (CE) of 30 V. Gas 1, Gas 2, and Curtain Gas were 60 Psi, 60 Psi and 30 Psi, respectively, source temperature was 600°C with 5,000 V and -4,000 V of ion spray voltage floating (ISVF) for the positive and negative mode respectively, were set as the ESI sources conditions.

### Metagenomic Shotgun Sequencing

All samples were paired-end sequenced on an Illumina NovaSeq 6000 platform (insert size 350 bp, read length 151 bp) at Novogene Bioinformatics Technology. The microbial DNA was extracted by CTAB method. Adapter and low-quality reads were discarded, and the cleaned reads were filtered from human host DNA based on the human genome reference (hg19) as previously described (Qin et al., 2012). Next, 3,511.15 Gb of high-quality pair-end reads were acquired from 221 human gut microbiome samples with an average of 15.89 Gb per sample.

### Statistical Analysis

Unless stated otherwise, all statistical analyses were conducted using the R and the SPSS 21.0 software (SPSS Inc., Chicago, IL, USA). The clinical characteristics of participants enrolled in the study were expressed as mean ± standard deviation and the difference between groups was evaluated using one-way analysis of variance (ANOVA).

The converted metabolomics data were analysed using the XCMS software (https://metlin.scripps.edu/) to calculate the normalised peak intensity, retention time, and exact mass. The matrix was further reduced by removing peaks with missing values (ion intensity = 0) in more than 50% samples to obtain consistent variables. The relative standard deviation (RSD) value of metabolites in the QC samples was set at a threshold of 30%, as a standard in the assessment of repeatability in metabolomics data sets (Dunn et al., 2011). Each retained peak was normalised to internal standard (IS) and the data were introduced to SIMCA14 (Umetrics, Sweden) for principal components analysis (PCA) and orthogonal partial least squares discriminant analysis (OPLS-DA) with permutation-test (n = 200). The variables with variable importance in project (variable importance in project, VIP > 1, unpaired two-tailed Wilcoxon rank-sum test P < 0.05 and fold change (FC) > 1.2 or FC < 0.83) were considered significant metabolites. Metabolomics pathway analysis (MetPA) was conducted using the online tool MetaboAnalyst with hypergeometric test for over-representation analysis and relative-betweenness centrality for pathway topology analysis (Chong et al., 2018).

Differential abundance of phyla, genera, and species between B-CS patients and healthy controls were tested using the unpaired two-tailed Wilcoxon rank-sum test. The linear discriminant analysis (LDA) effect size (LEfSe) method was used to identify the greatest differences among specific characteristics of intestinal microbiota (http://huttenhower.sph.harvard.edu/lefse/) with the LDA scores (log10) > 2.0 (Segata et al., 2011). The statistical significant differences between metabolic or microbiota profile and individuals’ phenotypes were tested using permutation multivariate analysis of variance (PERMANOVA) using Bray-Curtis distance, and the number of permutations was 999. Unless otherwise stated, P < 0.05 was considered statistically significant. To obtain the functional profile, the high-quality reads were aligned to the updated gut microbiome gene catalogue (Li et al., 2014) using SOAP2 with a threshold of more than 90% identity over 95% of the length. Sequence-based gene abundance profiling was performed as previously described (Li et al., 2014). Next, the relative abundances of Kyoto Encyclopaedia of Genes and Genomes (KEGG) orthologous groups (KOs) were summed up from the relative abundance of their respective genes. Differentially enriched KEGG pathway/modules were identified according to their reporter score (Patil and Nielsen, 2005; Feng et al., 2015) from the Z-scores of individual KOs. A reporter score of ≥ 1.96 (95% confidence according to normal distribution) was used as a detection threshold for significantly differentiating pathways. The R language was used for microbiome data analysis and the database for taxonomy annotation is metaphlan2, besides, to annotate function, reads were mapped to IGC (Intergrated Gene Catalog) to gain gene abundance, then KEGG(Kyoto Encyclopedia of Genes and Genomes) database was used to annotate KEGG function.

Random forest (RF) analysis was used to screen potential metabolic and microbiota biomarkers for B-CS. RF models were used to rank each type of profiles independently and validated by 10-fold stratified cross-validation testing with default parameters in RF function of R package RF (ntree = 1,000). The optimal number of discriminatory markers for each type of profiles was determined using the recursive feature elimination method with default parameters using five different random seeds. The risk probability of B-CS disease for each subject was computed by the selected metabolic or microbiota features, and receiver operating characteristic (ROC) curves were constructed using pROC (Robin et al., 2011). The optimised RF model was further tested on the test datasets. Besides, Spearman’s rank-order correlation was used to determine the strength and direction of the monotonic relationships among variables [metabolites, gut microbiota (phyla, genera and species), KOs, and pathways].

## Results

### General Characteristics of Patients With B-CS and Healthy Controls

We collected 214 B-CS patient samples and 41 healthy control samples from 2018 from the two units. The baseline information [average age, sex, and basal metabolic index (BMI)] of the patients and healthy controls were comparable. Specimens with less abundant samples or those that changed during storage were excluded. We performed faecal metagenomics sequencing for 191 patients and 30 controls as well as serum metabolomics sequencing for 176 patients and 39 controls. We divided patients into untreated (n = 93) and treated (n = 121) groups based on whether invasive treatment (percutaneous recanalization) was performed at the time of sample collection. Simultaneously, according to our previous grouping pattern based on imaging findings and clinical manifestations (Fu et al., 2011), we subdivided the patients into four groups: IVCHT-PHT- (n = 17), IVCHT+PHT- (n = 23), IVCHT-PHT+ (n = 71), and IVCHT+PHT+ (n = 103) (**Table 1**).

**Table 1:** Clinical characteristics of patients with B-CS and healthy controls.

Characteristics	B-CS (n = 214)	Controls (n = 41)	P value
Number of participants with metabolite profiles	176	39	–
Number of participants with metagenomics profiles	191	30	–
Clinical characterization types			
IVCHT-PHT- (metabolite | metagenomics)	17 (12|16)	–	–
IVCHT+PHT- (metabolite | metagenomics)	23 (11|22)	–	–
IVCHT-PHT+ (metabolite | metagenomics)	71 (65|61)	–	–
IVCHT+PHT+ (metabolite | metagenomics)	103 (88|92)	–	–
Invasive treatment			
Yes (metabolite | metagenomics)	121 (96|108)	–	–
No (metabolite | metagenomics)	93 (80|83)	–	–
Age	46.31 ± 11.95	39.35 ± 12.50	0.6705
Gender (F/M)	88/128	26/14	0.0520
Body mass index (kg/m^2^)	21.43 ± 1.844	21.31 ± 1.580	0.6951
Serum bilirubin (umol/L)	29.89 ± 38.59	7.055 ± 2.476	0.0061
Serum albumin (g/L)	39.08 ± 6.489	44.79 ± 3.294	<0.0001
Prothrombin time (s)	14.05 ± 4.310	12.02 ± 0.7119	0.0033
Child-Pugh			
A (metabolite | metagenomics)	144(121|133)	40 (39|30)	
B (metabolite | metagenomics)	50 (41|43)	–	
C (metabolite | metagenomics)	20 (14|15)	–	

B-CS, Budd-Chiari syndrome; PHT, portal hypertension; IVCHT, inferior vena caval hypertension.

### Metagenomics Profile of B-CS

In this study, diversity was evaluated using the richness measure (number of microbial taxa with nonzero counts) and Shannon index. The α diversities at species and gene levels did not differ significantly among the groups (P > 0.05). Furthermore, the results of principal coordinate analysis (PCoA) for β diversity at species, phylum, genus, and gene levels significantly varied among groups (P > 0.05). We examined the differences in microbial composition of the different groups by decomposing the total variance based on the distance matrix. We found that the results of PERMANOVA analysis (Bray-Curtis distance, n = 999) was not significantly different: the P values for species, genus, phylum and genes were 0.694, 0.758, 0.548, and 0.543, respectively. (**Supplementary Figure 1**).

**Figure 1:**
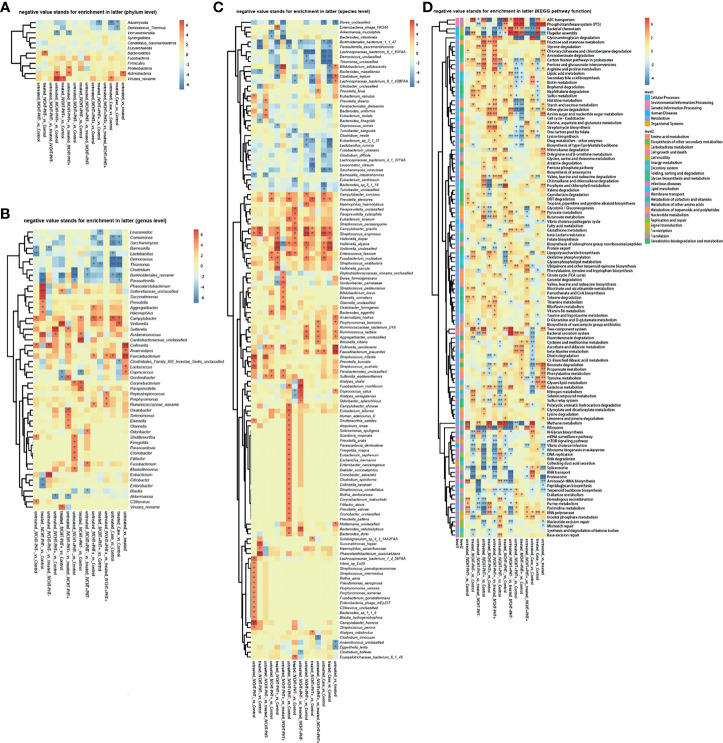
Faecal metagenomics profile in patients with B-CS: **(A)** Heat map of different phyla. **(B)** Heat map of different genera. **(C)** Heat map of different species. **(D)** KEGG pathway analysis of B-CS. Pathways involved in energy metabolism and substance transport, among others, were altered. *P < 0.05, **P < 0.01, enrichment direction as shown. B-CS, Budd-Chiari syndrome; KEGG, Kyoto Encyclopaedia of Genes and Genomes.

However, gut microbiota was different in untreated B-CS patients compared with that in the control group. The diversity varied as follows: at the phylum level, *Ascomycota* was significantly decreased (**Figure 1A**); at the genus level, *Campylobacter* was increased, while *Comamonas*, *Saccharomyces*, *Deinococcus*, *Thiomonas*, and *Burkholderiales noname* were decreased (**Figure 1B**); at the species level, *Prevotella stercorea*, *Campylobacter gracilis*, *Streptococcus anginosus*, *Veillonella atypical*, and *Veillonella unclassified* were increased, and *Burkholderiales bacterium_1_1_47*, *Lachnospiraceae bacterium_6_1_63FAA*, *Deinococcus unclassified*, *Thiomonas unclassified*, and *Saccharomyces cerevisiae* were decreased (**Figure 1C**). The results showed dysbiosis of gut microbiota in untreated B-CS.

To assess the functional consequences of changes in microbial communities, we performed enrichment analysis using Kyoto Encyclopedia of Genes and Genomes (KEGG) annotations. Compared to that in the healthy controls, twenty-four functions were reduced in the untreated B-CS patients, including phosphotransferase system (PTS), flagellar assembly, ABC transporters, benzoic acid degradation, nitrogen metabolism, galactose metabolism, bacterial chemotaxis, and lipopolysaccharide biosynthesis. Eighteen functions involving phosphoinositide metabolism, secondary bile acid biosynthesis, valine, leucine and isoleucine degradation, proteasome function, N-glycan biosynthesis, RNA transport, glycosaminoglycan degradation, and methane metabolism were enriched in untreated B-CS (**Figure 1D**). Overall, these results illustrated changes at the level of the microbial community structure, and functional potential.

### Alteration of Serum Metabolites in Patients With B-CS

**Figure 2:**
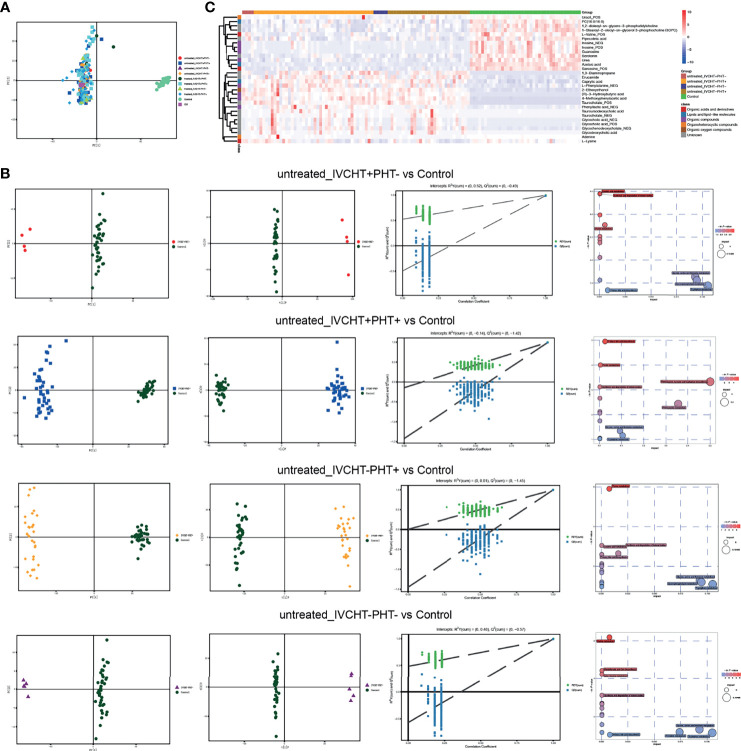
Alterations in serum metabolite levels in patients with B-CS: **(A)** PCA scores plot of data from a metabolomics scan of serum samples from B-CS patients with those from healthy controls. **(B)** Multivariate analysis of different untreated B-CS status (IVCHT+PHT-, IVCHT+PHT+, IVCHT-PHT+ and IVCHT-PHT- B-CS patients) with healthy controls. PCA and OPLS-DA scores plot with permutation-test (n = 200) clearly distinguished B-CS group from healthy controls. MetPA analysis highlighted the potential importance of distinct pathways that comprised the human metabolome that were represented by metabolites associated with B-CS. Each bubble present on pathway as well as the colour and size of each circle was based on P values and pathway impact values, respectively. **(C)** Heatmap of 30 significant metabolites in B-CS patients vs. healthy controls. (VIP > 1, q-value < 0.05, and fold change value > 1.2 or < 0.83.) B-CS, Budd-Chiari syndrome; KEGG, Kyoto Encyclopaedia of Genes and Genomes; PCA, principal components analysis; OPLS-DA, orthogonal partial least squares discriminant analysis; MetPA analysis, metabolomics pathway analysis; VIP, variable importance in project; IVCHT, inferior vena caval hypertension; PHT, portal hypertension.

Following pre-process, 1,866 peaks from positive mode and 810 from negative mode were log-transformed and merged for subsequent analyses. Three-dimensional data, including the sample name, peak number, and normalized peak area, were analysed using SIMCA 14 for principal components analysis (PCA) and orthogonal partial least squares discriminant analysis (OPLS-DA). The PCA scores plot distinguished the control group from the four B-CS groups (IVCHT+PHT-, IVCHT+PHT+, IVCHT-PHT+, and IVCHT-PHT-) (**Figure 2A**). Supervised OPLS-DA (7-fold cross validation) model was applied to better understand the variables responsible for classification of B-CS groups *vs*. healthy controls. The parameters for the classification were R2Y = 0.996 and Q2 = 0.985 (untreated IVCHT+PHT- *vs*. control groups), R2Y = 0.995 and Q2 = 0.989 (untreated IVCHT+PHT+ *vs*. control groups), R2Y = 0.996 and Q2 = 0.993 (untreated IVCHT-PHT+ *vs*. control groups), and R2Y = 0.998 and Q2 = 0.991 (untreated IVCHT-PHT- *vs*. control groups), which were stable and reliable for further prediction (**Figure 2B**). The four untreated B-CS groups also were clearly separated from the control group in the OPLS-DA scores plot (**Figure 2B**). These results indicated that B-CS was notably different from healthy controls at the serum metabolic level.

Compared with the serum metabolomics of controls, we identified 30 differential metabolites in untreated IVCHT+PHT-, IVCHT+PHT+, IVCHT-PHT+ and IVCHT-PHT- B-CS patients (**Figure 2C**). The levels of partially gut microbiota engaged synthesized metabolites, such as bile acids (e.g., taurocholate, glycocholic acid, glycochenodeoxycholate, tauroursodeoxycholic acid, and glycycodeoxycholic acid), short-chain aromatic fatty acid derivatives (e.g., (R)−3−hydroxybutyric acid), phenylalanine and derivatives (e.g., L−phenylalanine, phenyllactic acid, and 4-methoxyphenylacetic acid) were significantly increased, while purine metabolites (e.g., inosine, guanosine, and uracil), branch-chain amino acid (e.g., L-valine), tryptophan derivatives (e.g., serotonin) were significantly decreased in B-CS patients compared with that in healthy controls. These serum metabolites were significantly different at untreated B-CS, suggesting serum metabolites might be novel diagnostic biomarkers for the identification of B-CS and indicating there were sophisticated interactions among gut microbiota, serum metabolites and B-CS manifestations.

To understand the mechanism underlying B-CS, we further applied metabolomics pathway analysis (MetPA), and found that purine metabolism, ketone body synthesis and degradation, primary bile acid biosynthesis were significantly altered in untreated B-CS (**Figure 2B**). The results suggest that large scale pathway changes of metabolism were associated with the pathogenesis of B-CS.

### Comparison of Random Forest (RF) Models Based on Metagenomics and Metabolome Features

**Figure 3:**
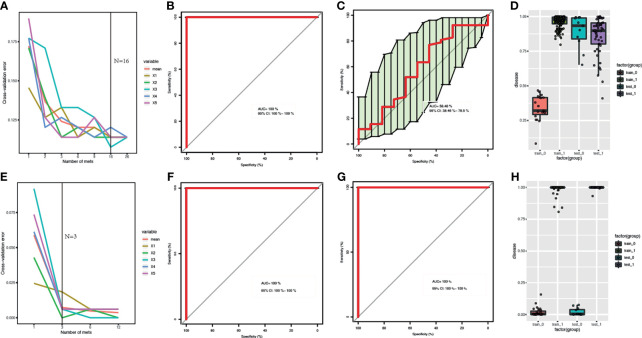
Comparison of RF models based on metagenomics and metabolome features: **(A, E)** Distribution of 5 trials of 10-fold cross-validation error in random forest classifier. The red solid curve showed the average of the 5 trials, the black solid line indicated the number of picked features in the optimal set. **(B, C, F, G)** ROC curve and AUC for the training and test datasets, respectively. **(D, H)** Box-and-whisker plot presented the risk probability of B-CS. **(A-D)** for B-CS faecal microbiota. **(E–H)** for B-CS serum metabolome. The metabolomics results showed that both the training datasets and test datasets performed perfectly with (AUC = 100 %, 95% CI: 100 % – 100 %) **(F–H)**. We found that metagenomics performed relatively poorly on test datasets (AUC = 58.48 %, 95 % CI: 38.46 % – 78.5 %) **(C, D)**, although the training datasets showed good performance (AUC = 100 %, 95 % CI: 100 % – 100 %) **(B–D)**. RF, random forest; B-CS, Budd-Chiari syndrome; RF, random forest; ROC curve, Receiver Operating Characteristics curve; AUC, area under the curve; CI, confidence interval.

The samples of The First Affiliated Hospital of Zhengzhou University were used as the training datasets, while the other was used as the test datasets. Training samples were used to obtain differential expressed metabolites and gut bacteria between B-CS patients and healthy controls to build RF models. For metagenomics, the sample size of training is 163, while the size of test is 52, while for metabolomics, the sample size of training is 158,while the size of test is 63. The optimized combinations of gut bacteria panel or metabolites panel were obtained (**Figures 3A, E**). The metabolomics results showed that both the training datasets and test datasets performed perfectly (area under the curve, AUC = 100%, 95% confidence interval, CI: 100% – 100%) (**Figures 3F–H**). We also found that metagenomics performed relatively poorly on test datasets (AUC = 58.48%, 95% CI: 38.46% – 78.5%) (**Figures 3C, D**), although the training datasets showed good performance (AUC = 100%, 95% CI: 100% – 100%) (**Figures 3B, D**). This indicates that the detection of serum metabolites is more advantageous for the identification of B-CSs.

In terms of variable importance, intestinal bacteria, including *Clostridium leptum* (mean decrease Gini = 3.505), *Coprobacillus_sp_29_1* (mean decrease Gini = 0.794), and *Parasutterella_excrementihominis* (mean decrease Gini = 2.572), were the most associated with B-CS. Among the metabolomic variables, the highest ranked variables were taurocholate (mean decrease Gini = 9.723), azelaic acid (mean decrease Gini = 8.143) and (R)-3-hydroxybutyric acid (mean decrease Gini = 6.734). These findings indicate that significantly different gut microbiome and serum metabolites might contribute to B-CS progress.

### *Campylobacter concisus* Is a Potential Diagnostic Biomarker for B-CS

**Figure 4:**
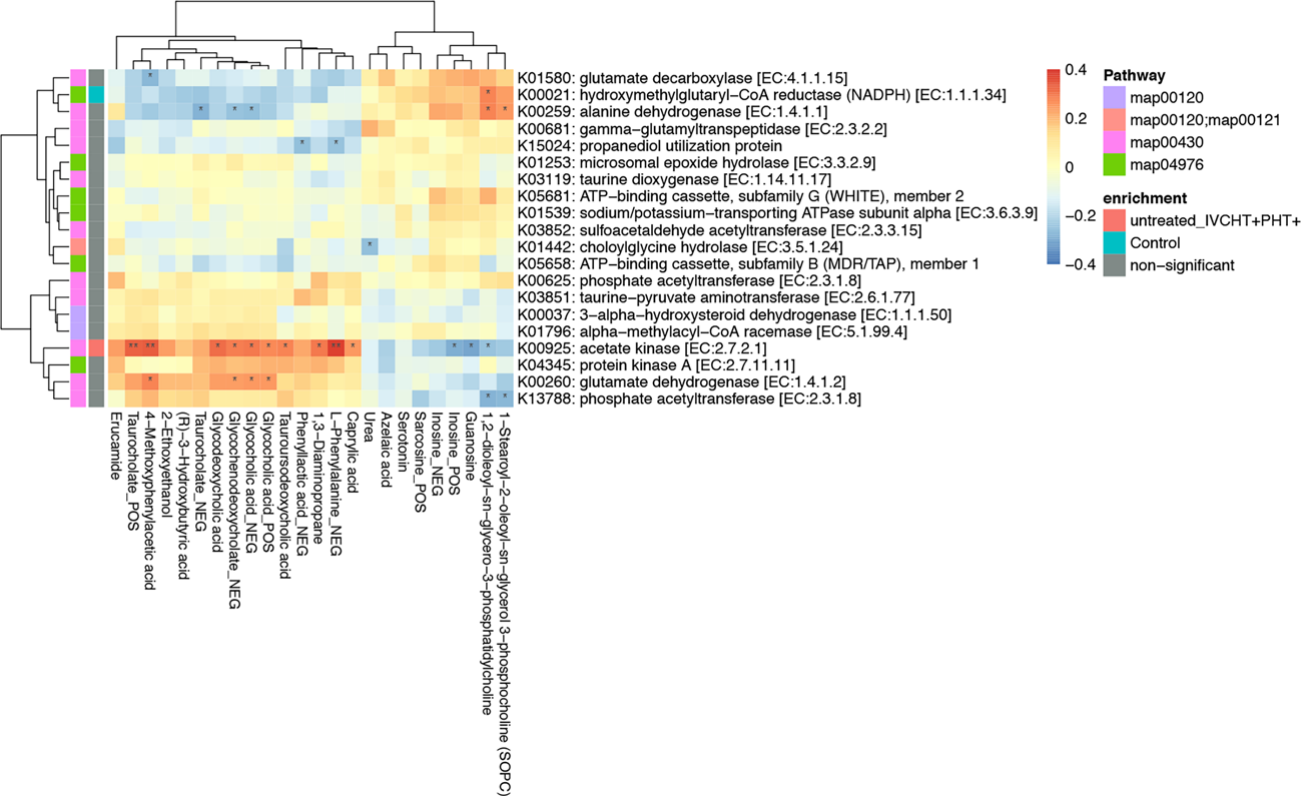
Taurocholate showed significant positive correlation with K00925: Acetate kinase [EC:2.7.2.1] of *Campylobacter concisus*. *P < 0.05, **P < 0.01, enrichment direction as shown.

We performed association analysis of intestinal bacteria and serum metabolites with phyla and species (**Supplementary Figure 2**). The results showed that in the presence of hypertension in untreated B-CS individuals, including those in IVCHT+ PHT-, IVCHT- PHT+, and IVCHT+ PHT+ groups, the *Campylobacter concisus* population significantly increased and showed a significant positive correlation with taurocholate. In particular, taurocholate showed significant positive correlation with K00925: acetate kinase [EC: 2.7.2.1] of *Campylobacter concisus* in the IVCHT+ PHT+ population (**Figure 4**). Further analysis of KEGG pathways revealed that taurocholate and dysregulated *Campylobacter concisus* are mainly associated with primary bile acid biosynthesis, fatty acid biosynthesis, and fatty acid metabolism, which are the key metabolic pathways involved in the development of liver diseases (**Supplementary Figure 3**). Thus, *Campylobacter concisus* may affect taurocholate in serum, which in turn is involved in the development of hypertensive symptoms in patients with B-CS.

### Status of IVCHT and PHT in B-CS

**Figure 5:**
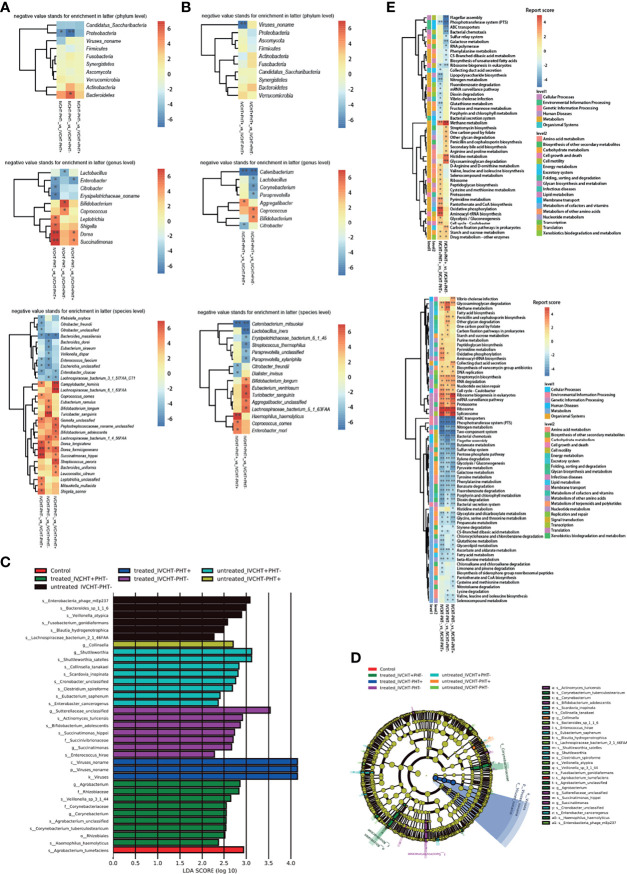
Status of IVCHT and PHT in patients with B-CS: **(A)** Heat map of different levels (phyla, genus and species) between IVCHT-PHT-patients and other patients in B-CS independent of treatment. **(B)** Heat map of different levels (phyla, genus and species) between IVCHT+PHT+ patients and IVCHT-PHT+/IVCHT+ PHT- patients in B-CS independent of treatment. **(C)** Distribution of greatest differences in taxa of B-CS vs. healthy controls using LEfSe analysis were presented according to the LDA scores (log_10_) > 2.0. **(D)** Evolutionary clade of B-CS different intestinal microbiota, as determined using the LEfSe method. **(E)** Altered microbial functions in KEGG pathway between IVCHT+PHT+ and IVCHT+PHT-/IVCHT-PHT+, IVCHT-PHT- patients and other B-CS syndrome (IVCHT-PHT+, IVCHT+PHT- and IVCHT+PHT+ patients). B-CS, Budd-Chiari syndrome; PHT, portal hypertension; IVCHT, inferior vena caval hypertension; LDA, linear discriminant analysis; KEGG, Kyoto Encyclopaedia of Genes and Genomes; LEfSe, linear discriminant analysis effective size. *P < 0.05, **P < 0.01.

The clinical manifestations of B-CS primarily originate from IVCHT and PHT. However, the IVCHT-PHT- group had no corresponding clinical manifestations because of the opening of collateral circulation. We compared the IVCHT-PHT- group with other B-CS groups; the metabolomics analysis showed no significant differences in metabolites between the groups (**Supplementary Table 1**). However, the results of metagenomics showed significant difference in gut microbiota levels between IVCHT-PHT- and IVCHT-PHT+ patients (PERMANOVA P = 0.049), and between IVCHT-PHT- and IVCHT+PHT- patients (PERMANOVA P = 0.020) (**Supplementary Table 2**). At the phylum level, *Proteobacteria* were significantly enriched in IVCHT-PHT+ and IVCHT+PHT- patients. At the genus level, *Lactobacillus* was significantly enriched in IVCHT+PHT- patients, while *Citrobacter* was significantly enriched in IVCHT-PHT+ patients. At the species level, *Bacteroides massiliensis* was significantly enriched but *Dorea formicigenerans* was significantly decreased in IVCHT-PHT+, IVCHT+PHT- and IVCHT+PHT+ patients. *Enterobacter cloacae* was enriched, while *Campylobacter hominis*, *Gemella unclassified* and *Succinatimonas hippei* were decreased in IVCHT-PHT+ and IVCHT+PHT+ patients. *Lachnospiraceae bacterium_3_1_57FAA_CT1* was prominently enriched but *Lachnospiraceae bacterium_1_4_56_FAA* was prominently decreased in IVCHT+PHT- and IVCHT+PHT+ patients (**Figure 5A**). The results demonstrate that further dysbiosis of the gut microbiota occurred with the emergence of hypertension in patients with B-CS.

To further investigate the relationship between B-CS hypertensive symptoms and intestinal microbiota dysbiosis, we compared patients with IVCHT+PHT+ with those with IVCHT-PHT+ and IVCHT+PHT-, respectively. At the phylum level, no significant difference was observed between the groups. At the genus level, *Aggregatibacter* was significantly enriched in patients with IVCHT+PHT+ than in patients with IVCHT-PHT+, *Coprococcus* and *Bifidobacterium* were significantly enriched in patients with IVCHT+PHT+ than in patients with IVCHT+PHT-. *Catenibacterium* was significantly decreased in patients with IVCHT+PHT+. At the species level, *Haemophilus haemolyticus*, *Coprococcus comes*, and *Enterobacter mori* were significantly enriched in patients with IVCHT+PHT+ than in patients with IVCHT-PHT+. Compared to that in patients with IVCHT+PHT-, *Bifidobacterium longum*, *Eubacterium ventriosum*, *Turicibacter sanguinis*, *Aggregatibacter unclassified*, and *Lachnospiraceae bacterium_5_1_63FAA* were significantly enriched in patients with IVCHT+PHT+. *Catenibacterium mitsuokai* was significantly decreased in patients with IVCHT+PHT+ (**Figure 5B**). To identify the microbiota associated with B-CS, we performed an intergroup gut microbiota analysis using with the linear discriminant analysis (LDA) effective size (LEfSe) method. The maximum difference in surtax was determined based on the LDA score (**Figure 5C**). At the same time, the microbial structure and main bacteria between the two were compared to draw an evolutionary branch diagram (**Figure 5D**). These results confirm that the composition of the intestinal microbiota of B-CS patients with different types of hypertension varied.

To understand the relationship more clearly, we performed KEGG functional prediction. Compared to those in IVCHT-PHT- patients, the pentose phosphate pathway, butanoate metabolism, glycolysis/gluconeogenesis, dioxin degradation, ABC transporters, glycine, serine and threonine metabolism related to carbohydrate, xenobiotics biodegradation and metabolism, membrane transport, and amino acid metabolism were significantly enriched in IVCHT-PHT+, IVCHT+PHT-, and IVCHT+PHT+ patients. Interestingly, compared to those in patients with IVCHT+PHT- and IVCHT-PHT+, pathways associated with methane metabolism, starch and sucrose metabolism, and drug-other metabolism and energy metabolism, carbohydrate metabolism, xenobiotics biodegradation and metabolism enzymes were significantly enriched in patients with IVCHT+PHT+ (**Figure 5E**). These findings further indicate that intestinal microbiota dysregulation deteriorates with disease progression.

### Evaluation of the Effectiveness of Invasive Treatment for B-CS

We performed a comparative analysis of the pre- and post-invasive treatments of patients with B-CS (IVCHT-PHT+, IVCHT+PHT- and IVCHT+PHT+). We found no significant differences in the results of metabolomics (**Supplementary Table 3** and **Figure 2A**) and metagenomics analyses (**Supplementary Table 4** and **Supplementary Figure 1**). However, certain differential bacteria were present in the intestinal microbiota of patients with B-CS. At the phylum level, *Verrucomicrobia* was significantly enriched in patients with IVCHT-PHT+ following treatment; *Actinobacteria* and *Proteobacteria* were significantly enriched in patients with IVCHT-PHT+ before treatment (**Figure 1A**). At the genus level, *Akkermansia* was significantly enriched in patients with IVCHT-PHT+ following treatment. *Olsenella*, *Oxalobacter*, *Gordonibacter*, *Collinsella*, *Sutterella*, *Eikenella*, and *Selenomonas* were significantly enriched in patients with IVCHT-PHT+ prior to treatment. Similarly, *Burkholderiales noname*, *Blautia*, and *Clostridium* were significantly enriched in patients with IVCHT+PHT- following treatment; *Fusobacterium* and *Odoribacter* were enriched in patients with IVCHT-PHT+ before treatment. *Faecalibacterium*, *Ruminococcaceae noname*, *Peptostreptococcus*, and *Porphyromonas* were significantly decreased in the IVCHT+PHT+ patients following treatment (**Figure 1B**). We observed that B-CS patients have high levels of beneficial bacteria, low levels of harmful bacteria, and improved gut microbiota following invasive treatment.

To determine the function of dysregulated bacteria, we performed a comparative analysis of untreated and treated B-CS patients using KEGG pathway analysis. Reduction in flagellar assembly, proteasome, peptidoglycan biosynthesis, *Vibrio cholerae* infection and pathogenic cycle, and terpenoid backbone biosynthesis were observed in treated patients with B-CS. At the same time, two-component system, propionate metabolism, phenylalanine metabolism, galactose metabolism, glyceride metabolism, fructose and mannose metabolism, amino sugar and nucleotide sugar metabolism, benzoate degradation, and PTS were decreased in treated patients with B-CS. Notably, flagellar assembly and terpenoid backbone biosynthesis were reduced in untreated patients compared to those in treated patients and healthy controls, while RNA polymerase and other glycan degradation were increased (**Figure 1D**). In addition, prior clinical data from treated-groups showed worse liver function during last invasive procedure (data not shown). These findings suggest that although current invasive treatments do effect the functional recovery of B-CSs with hyperbaric symptoms, some degree of intestinal dysbacteriosis persisted following B-CS treatment.

At the same time, we also focused on the pre- and post-treatment conditions of patients with B-CS in the IVCHT-PHT- group. Clinically asymptomatic individuals who did not require invasive treatment, for the time being, are partially administered invasive treatment. No significant differential bacteria were observed at the phylum level. At the genus and species levels, fewer discrepant microbes were detected compared to those in the other groups. This suggests that although invasive treatments improved the gut microbiota of the patients in the IVCHT-PHT- group, gut dysbiosis persisted following treatment.

## Discussion

In this study, we characterized the alterations in the gut microbiome and associated serum metabolome, and screened biomarkers for B-CS patients with non-invasive treatment with diagnostic potential for patients with B-CS represented by abundance of *Campylobacter concisus* and taurocholate. Furthermore, in individuals with different types of B-CS, we observed differences in the compositions of the intestinal microbiome, which provided potential strategies for the clinical management of B-CS. Our tracking of the B-CS treatment scenario provides a possible explanation for the problems associated with current invasive treatment strategies, namely, overtreatment and insufficient treatment.

The alterations in the gut microbiome in our study complement our results from those of a previous report on microbiota dysbiosis in B-CS (Sun et al., 2018). This trend might be explained by a combination of major alterations in the gut environment that may support the growth of several microbes. Intestinal microbial composition and metabolites are closely associated with liver diseases (Fu and Cui, 2017). As probiotics, *Lactobacillus* and *Saccharomyces* could reduce liver pathological damage by reducing inflammatory factors and increasing energy metabolism (Everard et al., 2014; Xu et al., 2019). However, *Faecalibacterium*, *Campylobacter*, and *Prevotella* were associated with the pathogenesis of non-alcoholic fatty liver disease (Iino et al., 2019), spotty liver disease (Gregory et al., 2018; Van et al., 2018), and liver inflammation (Lukens et al., 2014). In the present study, we also showed that related probiotics are depleted in the gut microbiome of patients with B-CS, while the microbiota that could induce disease development increased. This further confirms that B-CS is a hepatic vascular disease associated with dysregulation of the gut microenvironment and metabolism.

Diet strongly affects human health, partially by modulating the gut microbiome composition (Wu et al., 2011; Kaczmarek et al., 2017). Animal models of non-alcoholic fatty liver disease and hepatocellular cancer have indicated that diet induced the development of liver disease (Asgharpour et al., 2016). Notably, unlike our previous results (Sun et al., 2018), we did not observe any significant difference between the diversities of the gut microbiota of patients with B-CS and the controls, possibly due to the fact that the healthy controls we enrolled in this study were family members with similar dietary habits as patients with B-CS. These results emphasise the importance of diet in B-CS pathogenesis. Furthermore, this suggests that alteration of the gut microbiota in patients with B-CS is critical.

We identified 30 metabolites that significantly differed between patients with untreated B-CS and controls using liquid chromatography-mass spectrometry (LC-MS). Some of these metabolites are associated with thrombosis or/and liver-related diseases (Lin et al., 2017; Xu et al., 2017). Analogous metabolomic profiles could be excellent clinical diagnostic markers in similar diseases such as idiopathic PHT, which does not require multiple and invasive tests (Seijo et al., 2013; Seijo et al., 2016). Furthermore, the metabolic pathways involved in B-CS, such as primary bile acid biosynthesis, fatty acid biosynthesis, and fatty acid metabolism, are key metabolic pathways in liver diseases (Byrne, 2010; Alves-Bezerra and Cohen, 2017; Jiao et al., 2018; Ma et al., 2018). Thus, these metabolites and their related pathways shed light on the potential mechanism of B-CS pathogenesis.

We used metagenomics and metabolomics features for establishing RF classification models. RF-metabolomics provided perfect predictions on training and test datasets. However, RF-metagenomics performed well on training datasets but no better on test datasets, which indicated shotgun metagenomics would need further research in the other clinical centres. Besides, the RF model helped to select important intestinal bacteria, including *Clostridium leptum*, and *Parasutterella_excrementihominis*. *Campylobacter* colonizes the human oral cavity, and some strains can be transferred to the intestine, which play an important role in the development of inflammatory bowel disease (IBD), Barrett’s oesophagus, and gastroenteritis (Mukhopadhya et al., 2012; Kaakoush et al., 2015). Notably, *Campylobacter concisus* population was significantly elevated in patients with B-CS and correlated positively with taurocholate in serum, which is a potential marker for B-CS diagnosis. In addition, taurocholate also showed significant positive correlation with K00925: Acetate kinase [EC: 2.7.2.1] of *Campylobacter concisus* in the IVCHT+ PHT+ population. This confirms the diagnostic potential of *Campylobacter concisus* for B-CS, especially in IVCHT/PHT individuals.

We observed no significant difference in the metabolic profiles of the IVCHT- PHT- and other B-CS groups, although several intestinal microbes differed between these groups. Patients with various subtypes of B-CS showed significant differential bacteria in the gut microbiota. These results illustrate the presence of specific characteristics in these patients and inconsistent components between PHT and IVCHT patients regarding dysregulation of the gut microbiota, which may correct dysbiosis (Hernandez-Gea et al., 2019).

Microbiota dysbiosis partially improved following invasive treatment (Aron-Wisnewsky et al., 2019). *Lactococcus* has been used to prevent and treat various liver injuries and diseases (Bakari et al., 2016; Xu et al., 2019). In this study, the population of predominant probiotics such as *Lactococcus* significantly increased following treatment. This suggests that the current mainstream invasive measurements for B-CS are effective. In addition, prior clinical data from treated groups showed worsening of liver function during the last invasive procedure. However, the results showed higher abundance of harmful bacteria, including *Prevotella* and *Campylobacter*, in the gut microbiome. Usually, these patients have abnormal liver function or related clinical manifestations, which are together indicative of insufficient therapy. Twelve patients with B-CS developed liver cancer (data not shown) during follow-up after invasive treatment, indicating that further invasive treatment was necessary.

Stepwise management strategies for patients with B-CS were widely accepted (Sun et al., 2013; Khan et al., 2019). However, owing to the differences in the characteristics of patients with B-CS between Western and Asian countries (Qi et al., 2015), clinical management strategies have not been clarified. We observed that IVCHT- PHT- individuals did not show any significant differences in gut microbiota and metabolomics before and after treatment. These results were indicative of overtreatment for patients with complete compensation. However, compensatory status cannot be determined easily from collateral circulation. In our previous study, treatment indication was assessed using invasive methods to measure the pressure of inferior vena cava (IVC) and main hepatic veins (MHVs) (Fu et al., 2011). In the present study, decisions based on metagenomic and metabolomic features may replace the necessity of invasive methods. Therefore, B-CS can be managed as a chronic disease in clinical settings, similar to the management strategy for hepatitis B virus -associated cirrhosis (Liaw et al., 2012). However, disease progression and decompensation may still occur, in which case invasive treatment should be considered.

Our study has certain limitations. The study included a small sample size of some groups. Although our analysis was data-driven, it requires further validation using multi-centres. We applied a mouse model of faecal microbiota transplantation to further strength the relationship between B-CS syndrome, gut microbiota and metabolites, contributing to investigating the mechanism of B-CS. We are currently prospectively collecting DNA/RNA information and comprehensive medical records from patients with B-CS and related controls at regular intervals to determine the cause underlying intestinal microbiome alteration in B-CS.

To the best of our knowledge, for the first time, we have fully determined the characteristics of the altered gut microbiota and serum metabolite profiles of patients with B-CSs. *Campylobacter concisus* could be potentially used as a non-invasive diagnostic marker of B-CS. We believe that B-CS is not only a vascular disease, but also a microbial and metabolic disease. We further demonstrated the importance of IVCHT and PHT in the management of B-CS and put forward the view of undertreatment and overtreatment of the current invasive treatment strategies for B-CS, providing novel potential strategies for chronic disease management in clinical settings.

## Data Availability Statement 

The datasets presented in this study can be found in online repositories. The names of the repository/repositories and accession number(s) can be found below: https://www.ncbi.nlm.nih.gov/, PRJNA680829 https://www.ebi.ac.uk/metabolights/, MTBLS2319.

## Ethics Statement

The studies involving human participants were reviewed and approved by the Ethics Review Committee of Life Sciences, Zhengzhou University. The patients/participants provided their written informed consent to participate in this study.

## Author Contributions

All persons who meet authorship criteria are listed as authors. The project was designed by YSun and RZhu. JL, WW, RL, and QL managed the project. QL, HX, LZ, RZhang, ZL, PX, TB, ZW, GW, JR, DJ, YSong, LL, XM, and MZ contributed to acquisition of clinical samples, patients’ information and clinical data analyses. YSun designed the analysis. YSun, LZ, RZhang, and QL performed the data analysis. YSun and QL wrote the paper. All authors contributed to the article and approved the submitted version.

## Funding

The project was supported by the National Natural Science Foundation of China (No. 81870457, No. 81900558).

## Conflict of Interest

The authors declare that the research was conducted in the absence of any commercial or financial relationships that could be construed as a potential conflict of interest.

## Publisher’s Note

All claims expressed in this article are solely those of the authors and do not necessarily represent those of their affiliated organizations, or those of the publisher, the editors and the reviewers. Any product that may be evaluated in this article, or claim that may be made by its manufacturer, is not guaranteed or endorsed by the publisher.
