# Supplementary figures and images for: Alterations in Faecal Metagenomics and Serum Metabolomics Indicate Management Strategies for Patients With Budd-Chiari Syndrome
Source: Front Cell Infect Microbiol. 2021 Oct 21;11:730091. doi: 10.3389/fcimb.2021.730091 (PMC8567795; doi:10.3389/fcimb.2021.730091)

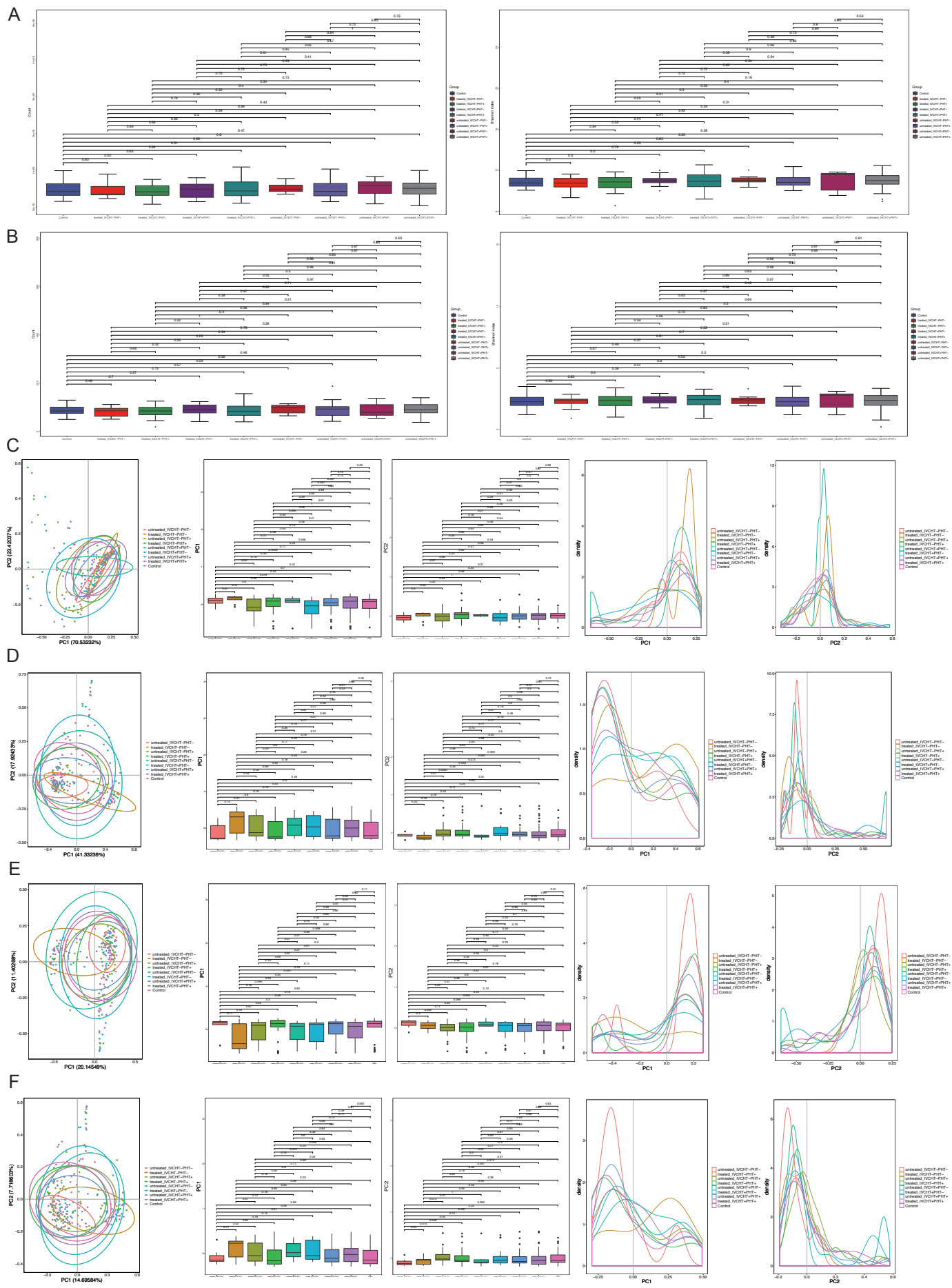

Supplement: Supplementary file 1 [file Image_1.pdf]

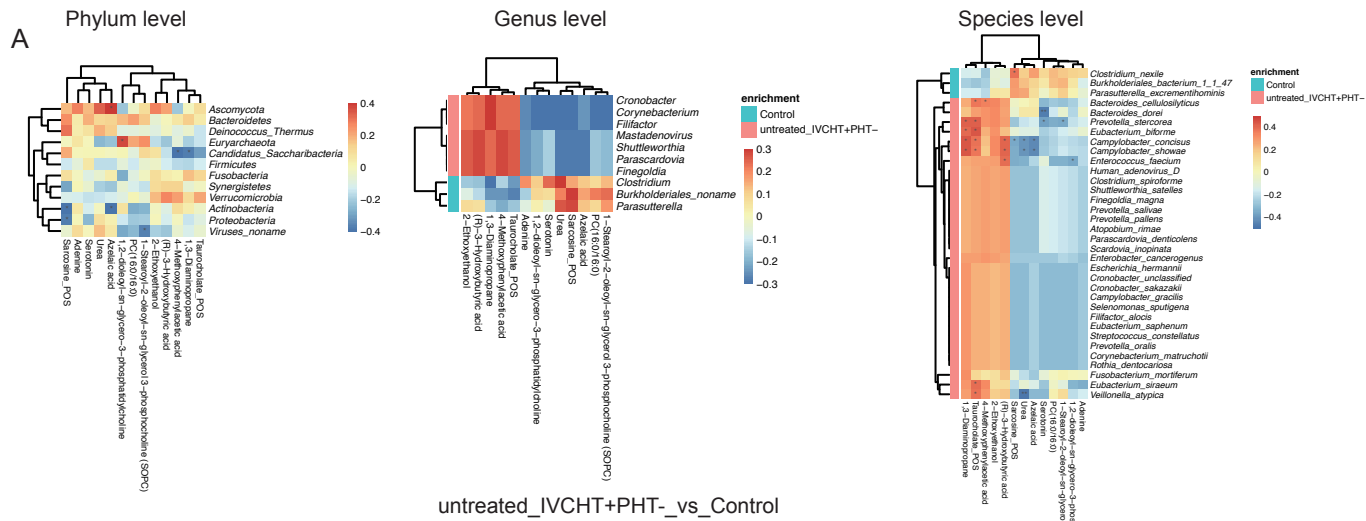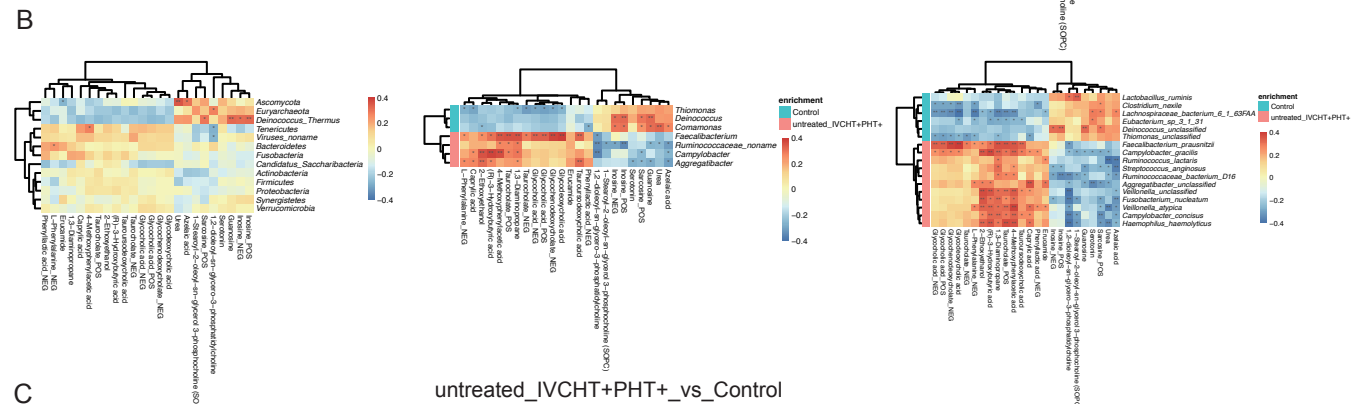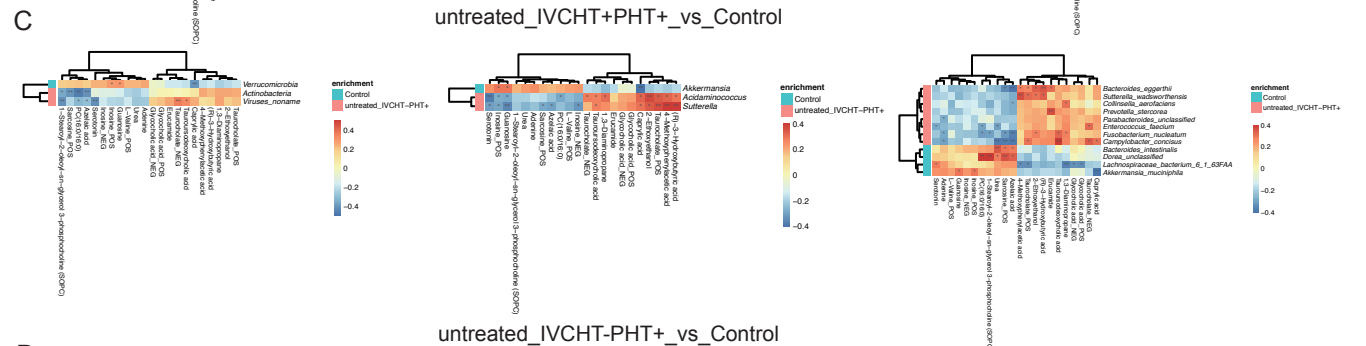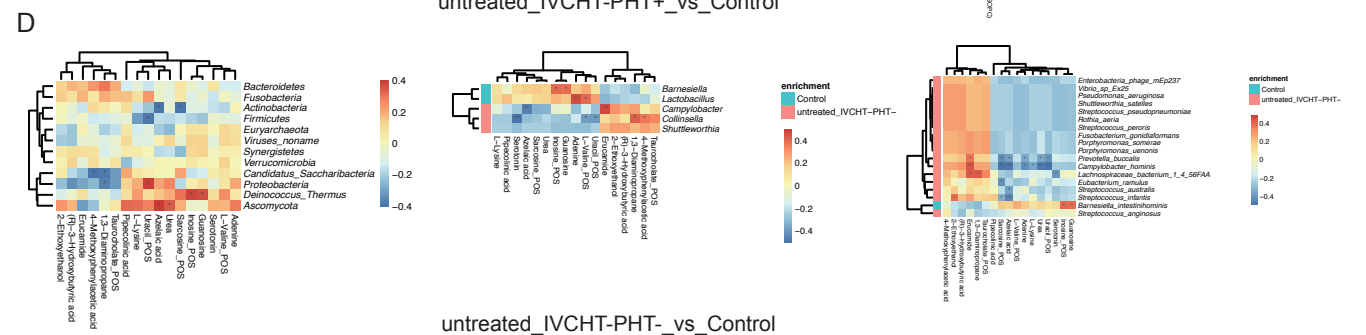

Supplement: Supplementary file 2 [file Image_2.pdf]
