# Supplementary material for: Alterations in Faecal Metagenomics and Serum Metabolomics Indicate Management Strategies for Patients With Budd-Chiari Syndrome
Source: Front Cell Infect Microbiol. 2021 Oct 21;11:730091. doi: 10.3389/fcimb.2021.730091 (PMC8567795; doi:10.3389/fcimb.2021.730091)
Supplement: Supplementary file 3 [file Image_3.pdf]

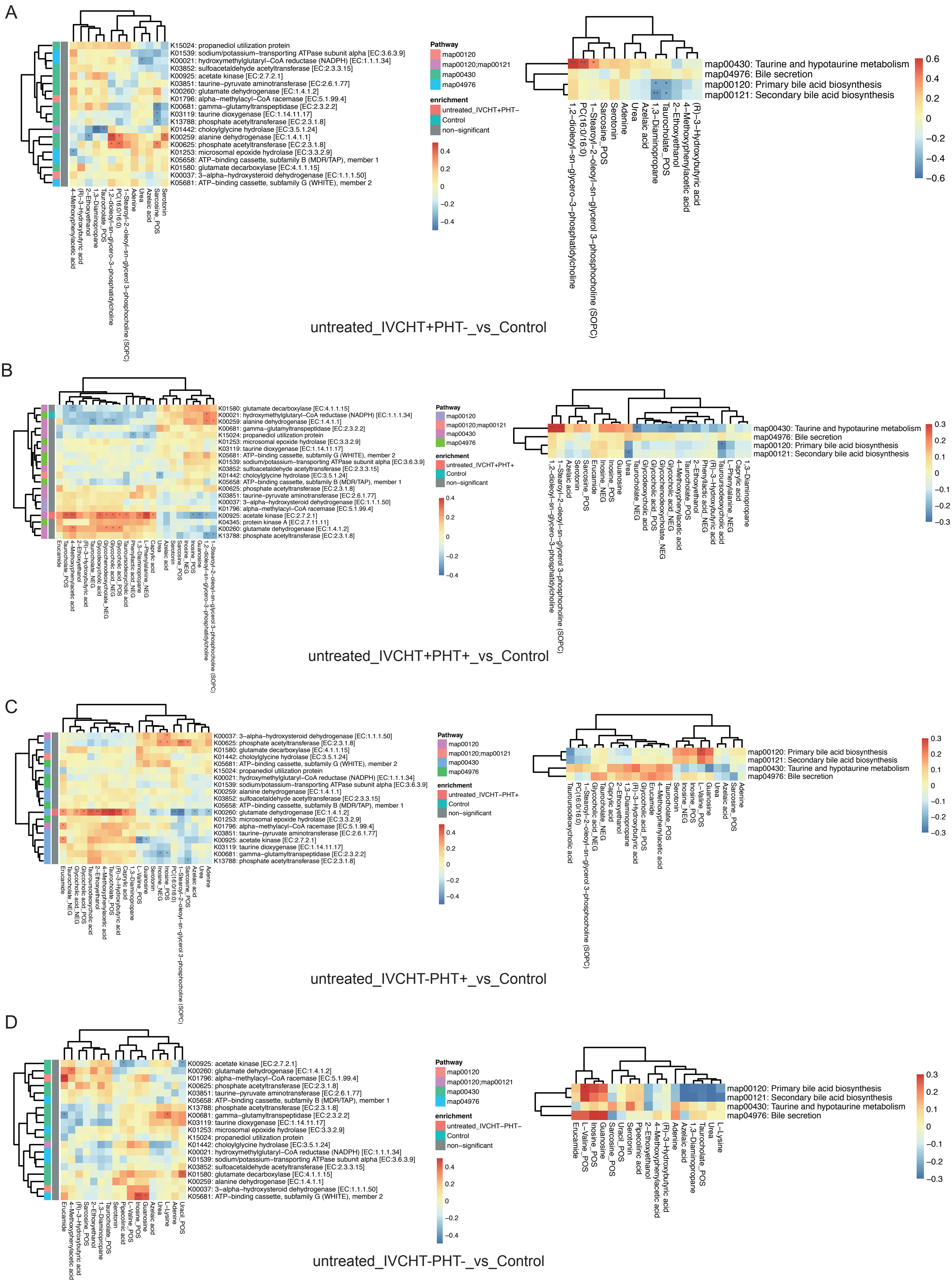
A
K15024: propanediol utilization protein
K01539: sodium/potassium−transporting ATPase subunit alpha [EC:3.6.3.9]
K00021: hydroxymethylglutaryl−CoA reductase (NADPH) [EC:1.1.1.34]
K03852: sulfoacetaldehyde acetyltransferase [EC:2.3.3.15]
K00925: acetate kinase [EC:2.7.2.1]
K03851: taurine−pyruvate aminotransferase [EC:2.6.1.77]
K00260: glutamate dehydrogenase [EC:1.4.1.2]
K01796: alpha−methylacyl−CoA racemase [EC:5.1.99.4]
K00681: gamma−glutamyltranspeptidase [EC:2.3.2.2]
K03119: taurine dioxygenase [EC:1.14.11.17]
K13788: phosphate acetyltransferase [EC:2.3.1.8]
K01442: choloylglycine hydrolase [EC:3.5.1.24]
K00259: alanine dehydrogenase [EC:1.4.1.1]
K00625: phosphate acetyltransferase [EC:2.3.1.8]
K01253: microsomal epoxide hydrolase [EC:3.3.2.9]
K05658: ATP−binding cassette, subfamily B (MDR/TAP), member 1
K01580: glutamate decarboxylase [EC:4.1.1.15]
K00037: 3−alpha−hydroxysteroid dehydrogenase [EC:1.1.1.50]
K05681: ATP−binding cassette, subfamily G (WHITE), member 2
Pathway
map00120
map00120;map00121
map00430
map04976
enrichment
untreated_IVCHT+PHT−
Control
non−significant
map00430: Taurine and hypotaurine metabolism
map04976: Bile secretion
map00120: Primary bile acid biosynthesis
map00121: Secondary bile acid biosynthesis
1,2−dioleoyl−sn−glycero−3−phosphatidylcholine
PC(16:0/16:0)
1−Stearoyl−2−oleoyl−sn−glycerol 3−phosphocholine (SOPC)
Sarcosine_POS
Serotonin
Adenine
Urea
Azelaic acid
1,3−Diaminopropane
Taurocholate_POS
2−Ethoxyethanol
4−Methoxyphenylacetic acid
(R)−3−Hydroxybutyric acid
untreated_IVCHT+PHT−_vs_Control
B
K01580: glutamate decarboxylase [EC:4.1.1.15]
K00021: hydroxymethylglutaryl−CoA reductase (NADPH) [EC:1.1.1.34]
K00259: alanine dehydrogenase [EC:1.4.1.1]
K00681: gamma−glutamyltranspeptidase [EC:2.3.2.2]
K15024: propanediol utilization protein
K01253: microsomal epoxide hydrolase [EC:3.3.2.9]
K03119: taurine dioxygenase [EC:1.14.11.17]
K05681: ATP−binding cassette, subfamily G (WHITE), member 2
K01539: sodium/potassium−transporting ATPase subunit alpha [EC:3.6.3.9]
K03852: sulfoacetaldehyde acetyltransferase [EC:2.3.3.15]
K01442: choloylglycine hydrolase [EC:3.5.1.24]
K05658: ATP−binding cassette, subfamily B (MDR/TAP), member 1
K00625: phosphate acetyltransferase [EC:2.3.1.8]
K03851: taurine−pyruvate aminotransferase [EC:2.6.1.77]
K00037: 3−alpha−hydroxysteroid dehydrogenase [EC:1.1.1.50]
K01796: alpha−methylacyl−CoA racemase [EC:5.1.99.4]
K00925: acetate kinase [EC:2.7.2.1]
K04345: protein kinase A [EC:2.7.11.11]
K00260: glutamate dehydrogenase [EC:1.4.1.2]
K13788: phosphate acetyltransferase [EC:2.3.1.8]
Pathway
map00120
map00120;map00121
map00430
map04976
enrichment
untreated_IVCHT+PHT+
Control
non−significant
map00430: Taurine and hypotaurine metabolism
map04976: Bile secretion
map00120: Primary bile acid biosynthesis
map00121: Secondary bile acid biosynthesis
untreated_IVCHT+PHT+_vs_Control
C
K00037: 3−alpha−hydroxysteroid dehydrogenase [EC:1.1.1.50]
K00625: phosphate acetyltransferase [EC:2.3.1.8]
K01580: glutamate decarboxylase [EC:4.1.1.15]
K01442: choloylglycine hydrolase [EC:3.5.1.24]
K05681: ATP−binding cassette, subfamily G (WHITE), member 2
K15024: propanediol utilization protein
K00021: hydroxymethylglutaryl−CoA reductase (NADPH) [EC:1.1.1.34]
K01539: sodium/potassium−transporting ATPase subunit alpha [EC:3.6.3.9]
K00259: alanine dehydrogenase [EC:1.4.1.1]
K03852: sulfoacetaldehyde acetyltransferase [EC:2.3.3.15]
K05658: ATP−binding cassette, subfamily B (MDR/TAP), member 1
K00260: glutamate dehydrogenase [EC:1.4.1.2]
K01253: microsomal epoxide hydrolase [EC:3.3.2.9]
K01796: alpha−methylacyl−CoA racemase [EC:5.1.99.4]
K03851: taurine−pyruvate aminotransferase [EC:2.6.1.77]
K00925: acetate kinase [EC:2.7.2.1]
K03119: taurine dioxygenase [EC:1.14.11.17]
K00681: gamma−glutamyltranspeptidase [EC:2.3.2.2]
K13788: phosphate acetyltransferase [EC:2.3.1.8]
Pathway
map00120
map00120;map00121
map00430
map04976
enrichment
untreated_IVCHT−PHT+
Control
non−significant
map00120: Primary bile acid biosynthesis
map00121: Secondary bile acid biosynthesis
map00430: Taurine and hypotaurine metabolism
map04976: Bile secretion
untreated_IVCHT−PHT+_vs_Control
D
K00925: acetate kinase [EC:2.7.2.1]
K00260: glutamate dehydrogenase [EC:1.4.1.2]
K01796: alpha−methylacyl−CoA racemase [EC:5.1.99.4]
K00625: phosphate acetyltransferase [EC:2.3.1.8]
K03851: taurine−pyruvate aminotransferase [EC:2.6.1.77]
K05658: ATP−binding cassette, subfamily B (MDR/TAP), member 1
K13788: phosphate acetyltransferase [EC:2.3.1.8]
K00681: gamma−glutamyltranspeptidase [EC:2.3.2.2]
K03119: taurine dioxygenase [EC:1.14.11.17]
K01253: microsomal epoxide hydrolase [EC:3.3.2.9]
K15024: propanediol utilization protein
K01442: choloylglycine hydrolase [EC:3.5.1.24]
K00021: hydroxymethylglutaryl−CoA reductase (NADPH) [EC:1.1.1.34]
K01539: sodium/potassium−transporting ATPase subunit alpha [EC:3.6.3.9]
K03852: sulfoacetaldehyde acetyltransferase [EC:2.3.3.15]
K01580: glutamate decarboxylase [EC:4.1.1.15]
K00259: alanine dehydrogenase [EC:1.4.1.1]
K00037: 3−alpha−hydroxysteroid dehydrogenase [EC:1.1.1.50]
K05681: ATP−binding cassette, subfamily G (WHITE), member 2
Pathway
map00120
map00120;map00121
map00430
map04976
enrichment
untreated_IVCHT−PHT−
Control
non−significant
map00120: Primary bile acid biosynthesis
map00121: Secondary bile acid biosynthesis
map00430: Taurine and hypotaurine metabolism
map04976: Bile secretion
Erucamide
L−Valine_POS
Inosine_POS
Guanosine
Sarcosine_POS
Uracil_POS
Serotonin
Pipecolinic acid
2−Ethoxyethanol
4−Methoxyphenylacetic acid
(R)−3−Hydroxybutyric acid
Adenine
Azelaic acid
1,3−Diaminopropane
Taurocholate_POS
Urea
L−Lysine
untreated_IVCHT−PHT−_vs_Control
