# Supplementary material for: Alterations in Faecal Metagenomics and Serum Metabolomics Indicate Management Strategies for Patients With Budd-Chiari Syndrome
Source: Front Cell Infect Microbiol. 2021 Oct 21;11:730091. doi: 10.3389/fcimb.2021.730091 (PMC8567795; doi:10.3389/fcimb.2021.730091)
Supplement: Supplementary file 4 [file Image_4.pdf]

Supplemental Figures Legends

Supplementary Figure 1 Diversity of B-CS in metagenomics analysis: (A) The number of observations (count) was used to indicate richness and the Shannon index was used to indicate homogeneity. Alpha diversity was not significantly different between patients with B-CS and healthy controls at the gene level ($P > 0.05$). (B) Alpha diversity was not significantly different between patients with B-CS and healthy controls at the species level ($P > 0.05$). (C) At the phylum levels, beta diversity was not significantly different between patients with B-CS and healthy controls ($P > 0.05$). (D) At the genus levels, beta diversity was not significantly different between patients with B-CS and healthy controls ($P > 0.05$). (E) At the species levels, beta diversity was not significantly different between patients with B-CS and healthy controls ($P > 0.05$). (F) At the genes levels, beta diversity was not significantly different between patients with B-CS and healthy controls ($P > 0.05$). B-CS, Budd-Chiari syndrome.

Supplementary Figure 2 Heat map of correlation analysis between each group of untreated patients with B-CS and healthy controls at phylum, genus, and species level in metagenomics analysis and serum metabolite levels. Significant spearman correlations were shown with * ($P < 0.05$) and ** ($P < 0.01$), enrichment direction is as shown.

B-CS, Budd-Chiari syndrome

Supplementary Figure 3 Heat map of correlation analysis between each group of untreated B-CS patients and healthy controls at metabolic pathway level and KO level. Taurocholate shows significant positive correlation with Campylobacter concisus in the presence of hypertension. Taurocholate also correlated significantly and positively with K00925: Acetatekinase [EC: 2.7.2.1] of Campylobacter concisus in the IVCHT+PHT+ population. Significant spearman correlations were shown with * ($P < 0.05$) and ** ($P < 0.01$), enrichment direction as shown.

B-CS, Budd-Chiari syndrome; PHT, portal hypertension; IVCHT, inferior vena caval hypertension; KO, Kyoto Encyclopaedia of Genes and Genomes (KEGG) orthologous groups
